# Periscope of Dermatology

**Published:** 1899-12-09

**Authors:** 


### PERISCOPE OF DERMATOLOGY.

**Lupus.**—Nelaton[1] strongly advocates the treatment of lupus by complete excision, combined with Thiersch's skingrafts. So far, he has seen no recurrences after this treatment, and the scar has been supple and in every way satisfactory. Brocq, Besnier, and Fournier are also inclined to agree with this method of treatment provided no evidence of internal tubercle exists.

Macleod,[2] in a paper on the modern treatment of lupus, describes the methods adopted at the Licht institut at Copenhagen for treatment by Finsens chemical rays. The leading principle in Finsens' apparatus is the exclusion of the heat rays, which is accomplished by passing the light through a cylinder containing a solution of sulphate of copper. The patients are under treatment for a quarter of an hour each day for about four to six months. The resulting scar is very good. He also refers to the hot air treatment, in which air at a temperature of 300° C. is pumped on to the lupus patch, producing an eschar, the lupoid tissue becoming charred before the healthy skin. The main difficulty is to ensure the complete destruction of the lupus without burning the unaffected part. He is inclined to recommend treatment by complete excision, combined with the immediate transplantation of a skin flap, consisting of the whole thickness of the skin. Finally, he describes Unna's wood spicule method; this consists in transfixing each nodule with a chemically-prepared spicule, which is a piece of hard wood pointed at both ends, and soaked for a few days in a strong mercury and carbolic solution. One of the pointed ends is inserted into a nodule and cut off, about six being inserted at a sitting without causing pain. The whole patch, with the spicules sticking out, is then covered with a piece of mercury and carbolic plaster, and left for two days; the spicules will then be found lying loose in little pustules. A simple ointment will heal the resulting sore.

Whitehouse[3] reports a severe case of lupus erythematosus in a woman, aged 52, treated by the internal administration of iodoform, in the form of a 0·06 gr. pill taken at meals. There was no improvement for the first three weeks, but it then commenced, and the case was cured after three months' treatment.

Reichel[4] records four cases of lupus erythematosus cured by the internal use of quinine during from 15 days to three months.

Hebra[5] recommends the following lotion to be used frequently in the form of a compress in lupus erythematosus: ℞. alcohol, ether; spirit menthæ piperitæ a.a.

Lawrence[6] reports three cases of lupus erythematosus successfully treated by linear scarification, and the application of india-rubber pressure pads. First, he performs superficial scarification, about 400 to the inch, then he rubs in iodoform, and the pressure pad is applied for four days. General anæsthesia is usually employed. No scar formation or disfigurement results. The author has also found the treatment of use in keloid.

**Therapeutics.**—*Naftalan:* Rothleder[7] arrives at the following conclusion about the use of naftalan: (1) It is a good remedy in the treatment of all eczema, except when accompanied with severe inflammation, being most useful in so-called industrial eczema; (2) it can be used in psoriasis; (3) it is a reducing agent equalling ichthyol, sulphur, &c., and can be tried where these are indicated. *Naphthalin:* Acholediani[8] describes the results of the treatment of 43 cases of eczema with this drug. It is applied as a 10 per cent. ointment. Once a day the ointment is washed off with soap and water, and fresh applied. The pain and itching have usually disappeared by the end of the first week, and the patches are completely healed within 38 days. *Tannoform:* Ehrmann[9] found good results to follow the employment of a 10 per cent. ointment of tannoform in cases of eczema, hyperidrosis, &c.; it was especially useful in eczema of the perinæum and between the toes. *Resorbin:* Ledermann[10] strongly recommends resorbin as an unguent base; it is made from an emulsion of pure almond oil and wax in water, and can be mixed with any vegetable, mineral, or animal grease; it has great power of penetrating the skin, and is suitable in all cases where the skin is to be quickly and thoroughly greased, or when it is desired to introduce a remedy into, or through the skin into the body in a vigorous manner. *Captol:* Eichhoff[11] recommends captol in seborrhœa. It is the product of the condensation of chloral and tannin, and is in the form of a white powder, which is soluble in hot water and in alcohol. The following formula is recommended: ℞. Captol, chloral hydras, acid tartaric, a.a. 1 part; castor oil, ½ part; and spirits of wine, 100 parts, fiat lot. It assuages itching, and prevents the falling out of the hair.

Freund[12] considers that the X rays are of therapeutic value in the treatment of lupus and in the removal of superfluous hairs. To cause epilation it is necessary to use a current of 2 ampères and a voltage of 11½ volts, the vacuum tube being about 10 inches from the part, and the sitting must not exceed 10 minutes in duration; a satisfactory result is obtained after from 20 to 30 sittings. In one case the hair became snow-white during treatment. In lupus the visible nodules first become red, then separate, leaving depressions, which later become dark red scars; so far the after history is satisfactory.

**Tumours.**—Nankivell[13] records the case of a coloured girl who had a large fibroma attached to each ear, and a rope of fibromata, an inch in thickness, extending the whole length of the sternum. She also developed a fibroma wherever she had been cut, over 100 of these being counted. The nature of the growths was evidently scar-keloid.

Aitken[14] records the case of a woman, aged 43, who for 13 years had suffered from small painless lumps on the forehead, scalp, and ears; there were about 30 in all, besides some small ones scattered over the trunk; they varied in size from a mere papule to as large as a cherry, the largest being two inches long and one inch wide. About 12 of the largest were removed, when it was found that they were adherent to the pericranium. On microscopic examination they were found to be adenomatous.

Purdon[15] recommends the following simple treatment for warts: If on the finger, a tight indiarubber finger-stall should be worn night and day; and if on the extremities, an indiarubber bandage should be tightly applied. In about six weeks the warts will have disappeared.

Danlos[16] reports the case of a woman in whom an epithelioma developed in the site of a nævus of the neck; this was removed and did not recur, but a sclerodermic zone with a typical lilac ring developed round the scar, and, on microscopic examination of a portion, proved to be an epithelioma which had developed in the hair follicles.

Politzer,[16] in a paper on the nature of xanthoma, comes to the following conclusions: (1) Xanthoma palpebrarum vulgare is not a neoplasm; it is the product of the degeneration of embryonically misplaced muscle fibres; (2) it bears no histological relationship to xanthoma tuberosum disseminatum; (3) the common xanthoma tuberosum and xanthoma diabeticorum are similar processes; (4) they are both connective tissue neoplasms—in both the cells undergo a fatty degeneration, resulting in the destruction of the cells and the ultimate more or less complete disappearance of the nodule, this being more rapid in the diabetic form; and

(5) generalised xanthoma is an irritative process whose cause must be sought in some systemic disturbance.

Mainzer[18] records a case of congenital elephantiasis which was neither of the dropsical lymphangectatic nor of the telangiectatic type, but which closely resembled true elephantiasis arabum as seen in the adult. The left upper, both lower extremities, and the external genitals were enormously enlarged; the X rays showed that there was no enlargement of the bones. The skin everywhere retained its normal colour and there was no trace of angiomata nor of fibromata; there was no disease of the heart, kidneys, or of the thyroid. There had been no history of renal disease, erysipelas, or elephantiasis in the family. The treatment recommended was systematic massage and elastic pressure on the affected parts.

Anderson[19] records the case of a native of Jamaica in whom multiple fibromata, cheloid, and elephantiasis were present at the same time. The interesting point in the case was that there was absolutely no etiological connection between these various diseases.

**Pemphigus.**—Luithlen[20] states that pemphigus neonatorum, though having no connection with ordinary pemphigus, can be communicated to older children and adults. The difference between the disease in babies and in adults is due to the horny layer of the skin in the latter offering greater resistance to the penetration of the micro-organism. This organism is culturally indistinguishable from the staphylococcus aureus, but when inoculated in the skin always produces a vesicle and never a pustule. He notes that an outbreak of pemphigus in a lying-in hospital commonly starts in a child born of a mother suffering from sepsis. As the vesicles are formed by the elevation of the horny layer alone, the disease is relatively benign, and easily cured as compared with adult pemphigus, where the malphigian layer is also involved. Pemphigus contagiosa would be a more suitable name. The treatment consists in avoiding baths, and applying a soothing ointment to the parts where the vesicles have burst and an antiseptic dusting powder to the rest of the body.

Spencer[21] records an outbreak of dermatitis exfoliativa neonatorum in a lying-in hospital. It was an infective dermatitis of sudden onset without any premonitory symptoms, showing special affinity for infants under three weeks in age and avoiding adults; it was usually unattended by constitutional symptoms, and presented a polymorphous eruption; it had a rapid course, and was followed by exfoliation. In cachectic children it was rapidly fatal. Out of 48 children born in the institution 23 contracted the disease and 5 died. The epidemic ceased after isolation of the affected children and their nurses, but reappeared when this precaution was relaxed.

**Drug Rashes.**—Hopf[22] records the following cases: (1) dermatitis due to the primula obconica occurring in a gardener; (2) a man aged 40 rubbed oleum Laurii into his neck for hoarseness, a week later he had generalised dermatitis, which remained for about 14 days and then desquamated freely; (3) a man aged 40 used an ointment containing 10 per cent. of eugallol for psoriasis. No bad symptoms followed the first two applications, but after the third the psoriasis patches became surrounded by a bright red infiltrated zone which increased and gave rise to a generalised dermatitis; vesicles then appeared, and finally crusts, which on separating left superficial ulcers.

Taylor[23] reports the case of a boy, who, after taking quinine in two-grain doses for several days, developed a temperature of 102 deg. and a scarlatiniform eruption. The temperature fell the next day, but the rash lasted till the fourth day, when copious desquamation occurred. A year later, after the use of quinine again, a similar eruption occurred, and this was repeated after a month's interval when quinine was again given.

[1] Semaine Med., June 14, 189? p. 205. [2] Brit. Jour. of Derm., Sept., 1899, p. 341. [3] New York Med. Jour., Feb. 4, 1899; Brit. Med. Jour. Epit., July 22, 1899; Treatment, June 22, 1899, p. 258. [4] Ditto, ditto. [5] Ditto, ditto; Wein Med. Woch., Dec. 24, 1898. [6] Inter. Med. Mag., Aug., 1899, p. 607; Intercol. Med. Jour., May 20, 899. [7] The Therap., May 15, 1899, p. 128. [8] Voy. Med. Jour., July, 1898; Treatment, June 22, 18?9, p. 258. [9] Ditto, ditto, p. 261. [10] The Therap., July 15, 1899, p. 158. [11] Zeitsch. f Derm., No. 5, 1898; Les Noveaux Remedes, Sept. 8, 1899, p. 397. [14] Wein Med. Woch., Nos. 22 and 24, 1899; Brit. Jour. of Derm., Aug., 1899, p. 337. [1] Lancet, Aug. 19, 1899, p. 492. [14] Brit. Med. Jour., June 24, 1899, p. 1,533. [15] Dublin Jour. of Med. Sci., Aug. 1, 1899, p. 99. [6] Semaine Med., July 19, 1899, p. 247. [17] New York Med. Jour., July 15, 1899, p. 73. [18] Brit. Med. Jour. Ep., Sept. 2, 1899, No. 186; Deutz. Med. Woch., 25, July 6, 1899, p. 436. [19] Jour. of Trop. Med., July, 1899. [20] Brit. Med. Jour. Ep., Sept. 2, 1899, No. 170; Wein Klin. Woch., Jan. 26, 1899. [21] Aust. Med. Gaz., June 20, 1899, p. 225. [22] Brit. Jour. of Derm., Sept. 1899, p. 370; Derm. Centralb., Oct., 1898. [23] Int. Med. Mag., Aug. 1899, p. 607. [24] Virgin. Med. Semi-Monthly, May 12, 1899.

